# TransNet–SAM2: A Transformer–Foundation Model Framework for Prompt-Free Segmentation of White Blood Cells in Microscopic Blood Smear Images

**DOI:** 10.3390/diagnostics16111737

**Published:** 2026-06-04

**Authors:** Julius Bamwenda, Mehmet Siraç Özerdem, Orhan Ayyildiz, Veysi Akpolat, İrem Akpolat

**Affiliations:** 1 Electrical & Electronics Engineering Department, Engineering Faculty, Dicle University, 21280 Diyarbakır, Türkiye; hbamwenda@gmail.com; 2 Department of Internal Medicine-Hematology, Medical Faculty, Dicle University, 21280 Diyarbakır, Türkiye; orhanay21@hotmail.com; 3 Department of Biophysics, Medical Faculty, Dicle University, 21280 Diyarbakır, Türkiye; vakpolat@dicle.edu.tr; 4 Department of Nutrition and Dietetics, Memorial Private Diyarbakır Hospital, 21070 Diyarbakır, Türkiye

**Keywords:** white blood cell segmentation, digital pathology, transformer, foundation model, SAM2, weakly supervised learning, hematological analysis

## Abstract

**Background**: Accurate segmentation of white blood cells (WBCs) in peripheral blood smear images is a fundamental step in computational hematology, enabling downstream tasks such as classification, morphological assessment, and quantitative analysis. However, reliable segmentation remains challenging due to staining variability, complex cellular morphology, overlapping structures, and limited availability of high-quality annotations. **Aim and Methods**: The aim of this study is to develop a robust and fully automated segmentation framework for white blood cells (WBCs) in microscopic blood smear images, providing a reliable foundation for subsequent computational analysis. We propose TransNet–SAM2, a hybrid deep learning architecture that integrates hierarchical transformer-based feature extraction with a foundation-model-based decoder for prompt-free segmentation. Specifically, a Swin Transformer backbone is employed to capture multi-scale contextual representations, which are subsequently aligned and fused through a feature adaptation module. The fused features are directly injected into the SAM2 mask decoder, replacing conventional prompt-based conditioning and enabling fully automatic segmentation. In addition, a weakly supervised self-training strategy is incorporated to utilize partially annotated data, improving model generalization while reducing annotation requirements. The proposed framework is evaluated using a clinically curated dataset from Dicle University, the publicly available Raabin-WBC dataset, and an additional external leukemic blast validation dataset (ALL-IDB) to assess robustness under both routine and atypical hematological conditions. **Results**: TransNet-SAM2 achieved a Dice coefficient of 0.95 ± 0.01 and IoU of 0.90 on internal testing, significantly outperforming U-Net (0.89), Mask R-CNN (0.90), and SAM2 (0.92) (*p* < 0.05). In cross-dataset evaluation (Dicle training, Raabin-WBC testing), the framework maintained strong performance (Dice: 0.91, IoU: 0.84), demonstrating robustness to domain shifts. Ablation studies confirmed each component’s contribution, with the full model improving Dice by 6% over a CNN baseline. Qualitative analysis showed accurate boundary delineation even with cell overlap and background clutter. **Conclusions**: These findings indicate that the proposed framework provides a promising and scalable framework for WBC segmentation. While the current study focuses on segmentation, future work will investigate integration with classification and radiomics pipelines, as well as validation on more diverse clinical datasets, including bone marrow and leukemia samples.

## 1. Introduction

Microscopic examination of peripheral blood smears remains an essential component of modern hematological diagnosis. Morphological evaluation and differential counting of white blood cells (WBCs) provide important information for the diagnosis and monitoring of diseases ranging from acute infections to hematological malignancies such as leukemia [1,2]. In routine clinical practice, this analysis is usually performed manually, making the process labor-intensive, time-consuming, and prone to inter-observer variability and fatigue-related errors [3]. These limitations become even more significant when screening for rare but clinically important cells, such as blast cells in suspected acute leukemia, or when performing quantitative assessments that require accurate delineation of cell boundaries.

The development of whole slide imaging (WSI) has created new opportunities to address these challenges by converting conventional glass slides into high-resolution digital images suitable for computational analysis [1]. Digital pathology workflows have the potential to support human expertise through automated systems capable of producing more consistent and reproducible measurements across large numbers of cells. However, the integration of WSI technology into routine clinical hematology remains limited because of several important technical challenges. Microscopic blood smear images often show considerable variability caused by differences in staining protocols, illumination conditions, and slide preparation procedures [2]. In addition, the complex morphology of WBCs, including overlapping nuclei, irregular cytoplasmic boundaries, and dense surrounding erythrocytes, makes reliable automated segmentation particularly difficult [3].

It is important to clarify that, within the scope of this study, the term “segmentation” refers specifically to the pixel-level delineation of white blood cell boundaries in microscopic images. In clinical hematology, the same term may also refer to nuclear segmentation patterns in granulocytic leukocytes, which are outside the scope of the present work. Therefore, the proposed framework is intended as a preliminary computational tool that may support subsequent expert-guided classification and diagnostic evaluation of atypical or transitional cell populations.

### 1.1. Deep Learning for Medical Image Segmentation

The emergence of deep learning, particularly convolutional neural networks (CNNs), marked a significant advancement in biomedical image segmentation. Architectures such as U-Net introduced encoder–decoder frameworks with skip connections that effectively integrate contextual information with precise spatial localization [4]. Subsequent extensions incorporated attention mechanisms [5], nested skip pathways [6], and residual connections [7], to further enhance segmentation accuracy. Instance-aware frameworks, such as Mask R-CNN, extended these capabilities to handle overlapping cellular structures by jointly performing object detection and segmentation [8].

Despite these advances, CNN-based models remain constrained by their inherently local receptive fields, which limit their ability to capture long-range contextual relationships essential for resolving ambiguous boundaries in densely populated cellular regions. This limitation becomes clinically significant when distinguishing adjacent or overlapping cells in blood smears, where accurate delineation requires an understanding of spatial relationships between neighboring cells.

Transformer architectures address this limitation by modeling global dependencies through self-attention mechanisms. The Vision Transformer (ViT) demonstrated that images can be represented as sequences of patches, enabling contextual reasoning across the entire visual field [9]. Hierarchical variants, such as the Swin Transformer, further improved computational efficiency for high-resolution medical images [10], while hybrid architectures, including TransUNet and UNETR, combined the strengths of CNNs and transformers to achieve state-of-the-art performance in various medical segmentation tasks [11,12].

Nevertheless, these models typically require large volumes of annotated data, which poses a significant challenge in clinical settings where expert annotations are limited.

Accurate WBC segmentation is a critical first step for subsequent quantitative analysis. Without precise delineation of cell boundaries, later tasks such as feature extraction, classification, and cell counting may become unreliable [13]. Traditional image processing approaches, including thresholding, edge detection, and region-based methods, have shown limited effectiveness because they are highly sensitive to staining variability and often fail to generalize across diverse clinical samples [14].

### 1.2. Foundation Models and SAM-Based Segmentation

More recently, foundation models have introduced a major shift in computer vision research. This development has attracted considerable interest in adapting SAM-based architectures for medical image analysis. Approaches such as MedSAM [15], SAM-Adapter [16], Medical Transformer [17], SAM2 [18], TransFuse [19], and other pathology-oriented foundation-model adaptations [20,21,22]. The Segment Anything Model (SAM) demonstrated the possibility of a unified segmentation framework capable of generalizing across diverse visual domains through prompt-based interaction [20]. Nevertheless, many SAM-based medical segmentation methods still depend on prompt-guided interaction mechanisms, which may limit scalability in fully automated clinical workflows [20]. In addition, several existing adaptation strategies use relatively loose integration between feature extraction and mask generation modules [15,16,20,22]. As a result, these approaches may be less suitable for high-throughput digital pathology workflows where fully automated segmentation is required.

Most existing approaches therefore retain two important limitations that hinder their practical adoption in clinical settings. First, several adaptation strategies treat feature extraction and mask generation as relatively independent processes rather than as a tightly integrated framework [20,21,22,23,24]. Second, many SAM-based medical segmentation methods require explicit user prompts, such as points or bounding boxes, to initiate segmentation [20,21,25,26]. These limitations reduce their applicability in large-scale clinical workflows where manual intervention is impractical and consistent automated performance is essential.

Recent studies have highlighted the growing importance of transformer architectures in medical image segmentation, particularly for capturing long-range contextual dependencies and multi-scale representations. The Vision Transformer (ViT) [9] introduced global self-attention for image representation learning, while hierarchical transformer models such as the Swin Transformer [10] further improved computational efficiency for high-resolution medical images.

Unlike ViT, which applies global self-attention and exhibits quadratic computational complexity with respect to the number of image patches, the Swin Transformer uses a window-based self-attention mechanism that limits attention computation to local windows. This design reduces computational complexity from $0\left(N^{2}\right)$ to $0\left(N\cdot M^{2}\right)$, where N denotes the number of image patches and M represents the window size.

As a result, the Swin Transformer achieves a more efficient balance between computational cost and representation capability. Furthermore, its hierarchical architecture enables effective multi-scale feature extraction, which is particularly important for capturing both fine cellular details and broader contextual information in high-resolution microscopy images (e.g., 256 × 256 pixels or larger).

Based on these considerations, the Swin-Tiny variant is adopted as the backbone of the proposed TransNet framework because it provides an effective balance between computational efficiency and segmentation performance for digital pathology applications.

### 1.3. The Annotation Challenge

Another major challenge limiting the clinical adoption of automated segmentation methods is the scarcity of pixel-level annotations. Creating high-quality segmentation masks for white blood cells requires expert pathologists to manually delineate cellular boundaries, a process that is extremely time-consuming and difficult to perform on a large scale [27].

To address this limitation, weakly supervised and semi-supervised learning approaches, including pseudo-labeling and self-training methods, have been widely explored [28,29,30]. These approaches have demonstrated promising performance in histopathology and biomedical imaging applications [31,32,33]. However, in many studies, they are used mainly as auxiliary strategies rather than being fully integrated into the main segmentation framework.

More recently, several studies have investigated the combination of weak supervision with transformer-based and foundation-model architectures [34,35]. Despite these advances, the integration in most existing approaches remains relatively loose, which may reduce the overall effectiveness, consistency, and coherence of the learning process.

### 1.4. Positioning of the Proposed Method

Table 1 summarizes the design characteristics of existing segmentation approaches in relation to the proposed framework. CNN-based models, such as U-Net, enable fully prompt-free segmentation but have limited ability to capture long-range contextual dependencies and do not benefit from the generalization capability provided by foundation models. Transformer-based approaches, including TransUNet, improve global contextual modeling; however, they usually require large amounts of fully annotated training data.

Global Context refers to the ability to model long-range contextual relationships; Prompt-Free indicates segmentation without manual prompts or user interaction; Weak Supervision refers to the use of weakly supervised learning strategies; and Foundation Model indicates the utilization of a pre-trained foundation model architecture.

SAM-based adaptation frameworks, such as SAM-Adapter and SAM2-based segmentation approaches, leverage the strong representational capability of foundation models but often depend on external prompts for mask generation. In addition, many existing methods use relatively loose interaction between contextual feature extraction and segmentation decoding modules.

In contrast, the proposed TransNet–SAM2 framework combines transformer-based contextual modeling, prompt-free operation, weakly supervised learning capability, and foundation-model-based segmentation within a unified architecture.

The aim of this study is to develop a robust and fully automated framework for the primary computational segmentation and preliminary quantitative evaluation of white blood cells in microscopic blood smear images. In the present study, segmentation refers specifically to the pixel-level delineation of white blood cell regions within microscopy images, serving as an initial computational step for subsequent expert interpretation, hematological assessment, and diagnostic analysis.

The proposed framework is designed to support digital hematology workflows by assisting the identification, segmentation, and quantitative analysis of leukocyte regions in peripheral blood smear images, particularly in large-scale microscopy screening settings. In addition to routine leukocyte morphology, the framework is further evaluated using external leukemic blast-cell images to assess robustness under atypical hematological conditions.

Nevertheless, the proposed approach is not intended to replace expert manual diagnosis, flow cytometry, or comprehensive morphological evaluation of atypical hematological disorders. Instead, it is designed as a supportive computational tool that may assist future computer-assisted hematological analysis workflows.

### 1.5. Contributions

To address these challenges in preliminary computational leukocyte analysis, we propose TransNet–SAM2, a hybrid segmentation framework that combines transformer-based contextual feature learning with foundation-model decoding within a tightly integrated architecture. The proposed framework introduces several important contributions with emphasis on robustness, annotation efficiency, and practical applicability in computational hematology workflows.

**Prompt-free operation**: By directly integrating hierarchical transformer features with the SAM2 decoder, the framework enables fully automated segmentation without requiring manual prompts or user interaction.

**Cross-scale contextual alignment**: A feature integration mechanism preserves both global contextual relationships and fine-grained cellular details, thereby improving boundary delineation in morphologically complex microscopy regions.

**Annotation efficiency**: A weakly supervised self-training strategy is incorporated into the framework to reduce reliance on dense pixel-level annotations, addressing a major limitation in clinical dataset development.

**Comprehensive validation under heterogeneous hematological conditions**: The framework is evaluated using a clinically curated Dicle University dataset, the publicly available Raabin-WBC dataset, and an additional external leukemic blast validation dataset (ALL-IDB). These experiments include both routine leukocyte morphology and atypical blast-cell conditions, demonstrating improved robustness across heterogeneous staining, imaging, and morphological variations.

**Preliminary translational assessment**: In addition to segmentation experiments, a preliminary correlation analysis between automated segmentation-derived leukocyte quantification and expert manual differential counting is performed to provide initial insight into the agreement between the proposed framework and routine hematological assessment workflows.

By addressing key challenges in automated WBC segmentation, including manual prompting, limited cross-dataset generalization, and annotation scarcity, the proposed approach represents a step toward more reliable and scalable computational tools for digital hematology and microscopy-assisted analysis. Nevertheless, the framework is intended as a supportive computational system rather than a replacement for expert clinical diagnosis, flow cytometry, or comprehensive hematological interpretation.

It is important to distinguish between segmentation and classification tasks. Segmentation focuses on delineating cell boundaries at the pixel level, whereas classification assigns biological labels to cells. The present study addresses segmentation as a foundational computational step for subsequent quantitative analysis and expert interpretation.

The remainder of this paper is organized as follows: Section 2 describes the datasets and proposed methodology. Section 3 presents the experimental setup and results. Section 4 discusses the findings, clinical interpretation, and limitations of the proposed framework. Section 5 concludes the paper and outlines future research directions.

## 2. Materials and Methods

### 2.1. Datasets

To evaluate the proposed TransNet–SAM2 framework under both routine clinical and external validation conditions, three hematology-focused datasets are used in this study: a newly curated Dicle University dataset, the publicly available Raabin-WBC dataset [29], and the ALL-IDB acute lymphoblastic leukemia dataset. These datasets are selected to enable comprehensive assessment of segmentation performance across different imaging conditions, annotation protocols, leukocyte morphologies, and clinically atypical hematological presentations.

The Dicle University dataset is primarily used for model training and internal evaluation, while the Raabin-WBC dataset is employed for cross-dataset generalization analysis. In addition, the ALL-IDB dataset is incorporated for external validation on leukemic blast-cell morphology to evaluate the robustness of the proposed framework under atypical hematological conditions.

#### 2.1.1. Dicle University Dataset

The Dicle University dataset was collected in collaboration with the Departments of Pathology and Hematology at Dicle University Medical Faculty. All microscopic images were acquired using an Olympus CX18 optical microscope (Olympus Corporation, Tokyo, Japan) at 100× magnification under bright-field imaging conditions. Giemsa staining was applied to all blood smear samples to enhance cellular visibility and morphological detail. Variations in staining intensity and illumination were observed due to differences in slide preparation and image acquisition settings, reflecting realistic clinical variability. Ethical approval for the use of clinical data was obtained from the Dicle University Science and Engineering Ethics Committee (Approval No. 16/12/2021-194814, Approval date 31 December 2021).

The dataset consists of high-resolution WSIs obtained from routine clinical examinations. Because of the large size of WSIs, a patch-based processing strategy was adopted. A total of four WSIs were processed, from which 14,720 image patches of size 256 × 256 pixels were extracted. Patch extraction was performed using a sliding-window approach with overlap to ensure complete coverage of cellular regions.

To improve dataset quality, patches containing no relevant cellular structures or dominated by background regions were excluded using intensity-based and content-based filtering criteria.

In addition, image patches containing poor-quality or non-interpretable regions were excluded to ensure reliable model training and evaluation. Specifically, patches were removed if they contained out-of-focus regions where cellular boundaries could not be clearly identified, severe staining artifacts such as uneven dye precipitation or excessive staining, major illumination distortions including glare or non-uniform brightness, or motion blur affecting cellular structures. Patches with substantial cell truncation at image boundaries, extensive smear contamination or debris preventing reliable leukocyte identification, and patches containing only erythrocytes, platelets, or background regions without visible white blood cells were also excluded. These exclusion criteria were applied to improve annotation reliability and to simulate routine quality-control procedures commonly used in hematological microscopy analysis.

It is important to acknowledge that the application of these exclusion criteria, while necessary to ensure annotation reliability and training stability, may result in the removal of image patches containing rare or atypical cellular morphologies, including certain blast cell subpopulations with poorly defined boundaries or significant staining artifacts. Consequently, the dataset used for model training may not fully represent the complete spectrum of morphological heterogeneity encountered in routine clinical hematopathology, particularly for challenging leukemic blast forms. This limitation should be considered when interpreting the segmentation performance reported in this study, and future work should investigate the impact of less stringent exclusion criteria on model generalizability.

Pixel-level annotations were generated using the QuPath software v0.5.1 [36] by expert pathologists. Each white blood cell was manually delineated to produce accurate segmentation masks. The annotation process focused on three clinically relevant cell types: Blast, Neutrophil, and Lymphocyte. Background regions, including erythrocytes and smear artifacts, were labeled as non-target regions.

The blast-cell category includes immature leukocyte morphologies identified by expert hematologists from peripheral blood smear samples. Importantly, the dataset contains annotated blast cells together with normal neutrophils and lymphocytes. Therefore, the model is trained using both normal and atypical leukocyte morphologies from the beginning. The segmentation task does not involve classifying cells as benign or malignant; instead, it focuses on accurate delineation of cell boundaries regardless of pathological status. False-negative detection, corresponding to missed blast cells, is evaluated using the Recall metric, which remains high across the experiments. Nevertheless, the current dataset does not fully represent the complete spectrum of leukemic or highly atypical hematological morphologies encountered in clinical hematopathology. Therefore, the present study should be interpreted primarily as an investigation of automated segmentation performance rather than as a complete diagnostic evaluation framework for malignant hematological disorders.

Annotations were stored as binary masks aligned with the corresponding image patches, where foreground pixels represent white blood cells and background pixels correspond to non-cell regions. All image patches were stored in TIFF format with a resolution of 256 × 256 pixels, while the corresponding segmentation masks were saved as binary images with identical spatial dimensions.

In addition, annotation metadata, including bounding box coordinates and cell-type labels, were stored in JSON format to support structured data management and reproducibility.

The dataset includes both fully annotated and weakly annotated samples. Fully annotated samples contain pixel-level segmentation masks, whereas weakly annotated samples include coarse annotations such as bounding boxes or partial labels. Approximately 70% of the dataset consists of fully annotated samples, while the remaining 30% is used as weakly supervised data within the self-training strategy.

#### 2.1.2. Raabin-WBC Dataset

The Raabin-WBC dataset is a publicly available benchmark widely used for automated white blood cell analysis in deep learning studies involving cell classification, detection, and segmentation. The dataset contains high-resolution microscopic images of peripheral blood smears acquired under controlled laboratory conditions, together with expert-provided pixel-level annotations for multiple WBC subtypes, including lymphocytes, neutrophils, monocytes, eosinophils, and basophils [29].

The dataset originally contains 5000 high-resolution microscopic images (1920 × 1080 pixels) captured using an iPhone 7 camera mounted on a standard light microscope with a 20× objective lens. Images are stored in JPEG format with varying compression levels. To improve data quality, 120 poor-quality images containing severe focus problems or staining artifacts were excluded. Patch extraction was then performed using 256 × 256 pixel patches with a stride of 128 pixels, while patches containing less than 30% white blood cell area were filtered out. This process resulted in a final dataset of 11,200 image patches.

In this study, the Raabin-WBC dataset is used together with the proprietary Dicle University dataset to evaluate the robustness and cross-dataset generalization capability of the proposed segmentation framework. For consistency with the processing pipeline, all images were resized and divided into 256 × 256 pixel patches, enabling efficient training and improved learning of local cellular morphology.

**Figure 1:**
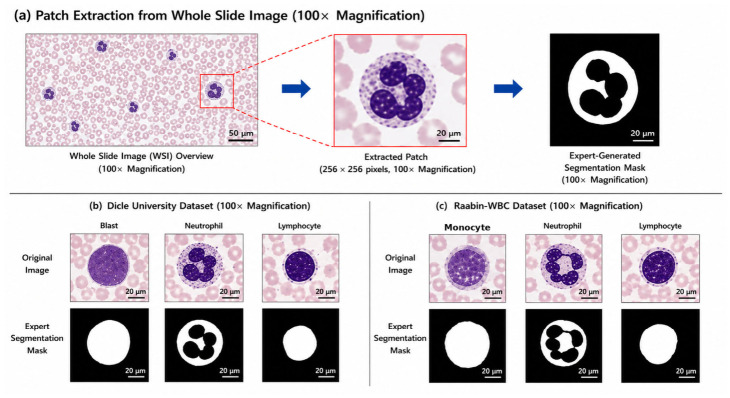
(**a**) shows a schematic of the patch extraction process, while panels (**b**,**c**) show real microscopic images with ground-truth annotations.

White blood cell regions were treated as the foreground class, whereas erythrocytes and background smear structures were retained as background. No dataset-specific parameter tuning was performed. Instead, preprocessing steps, training configurations, and model parameters were kept identical to those used for the Dicle University dataset to ensure fair evaluation of the TransNet–SAM2 framework under heterogeneous imaging conditions. Figure 1 illustrates representative image patches and corresponding segmentation masks from the datasets used in this study.

In (a), the patch extraction pipeline from a whole slide image (WSI) acquired at 100× magnification is illustrated. A region containing a white blood cell is selected and cropped into a 256 × 256 pixel image patch, followed by the generation of the corresponding expert-annotated segmentation mask. Scale bars indicate 50 μm at the WSI level and 20 μm at the patch level.

In (b), representative examples from the Dicle University dataset (100× magnification) are presented, including three clinically relevant cell types: Blast, Neutrophil, and Lymphocyte, together with their corresponding expert-generated segmentation masks.

In (c), representative examples from the Raabin-WBC dataset (100× magnification) are shown, illustrating different leukocyte morphologies, including neutrophils, lymphocytes, and monocytes, together with their corresponding expert-generated segmentation masks. All images shown are real microscopy images acquired either in our laboratory (Dicle University) or obtained from publicly available datasets. All segmentation masks are manually annotated by experts, ensuring high-quality ground truth for model training and evaluation.

The proposed patch-mask framework captures white blood cell morphology through pixel-level image representations extracted from microscopic blood smear patches. Although the framework does not explicitly use handcrafted cytological descriptors, relevant morphological characteristics are implicitly learned through deep feature extraction. These characteristics include nuclear size and shape, nucleus-to-cytoplasm relationships, staining intensity variations, cytoplasmic texture patterns, and granulation-related visual features observed across different leukocyte populations.

For example, neutrophils typically exhibit multi-lobed nuclear morphology and fine cytoplasmic granulation, whereas lymphocytes generally present with compact nuclei and limited cytoplasmic regions. Blast cells, including leukemic blast morphologies observed in acute lymphoblastic leukemia samples, often demonstrate enlarged nuclear regions, high nucleus-to-cytoplasm ratios, and relatively homogeneous chromatin appearance. Through transformer-based contextual feature learning and feature-level fusion with the SAM2 decoder, the proposed framework learns discriminative representations associated with these morphological patterns directly from microscopy images.

The inclusion of both routine peripheral blood smear samples and external leukemic blast-cell images enables evaluation of the proposed framework under both normal and atypical hematological conditions. These learned representations support robust computational delineation of white blood cell regions across heterogeneous staining, imaging, and morphological variations. Details of the datasets used in this study are summarized in Table 2.

The Dicle University dataset consists of peripheral blood smear images collected from routine clinical practice and annotated by expert pathologists. The Raabin-WBC dataset is a publicly available benchmark widely used for white blood cell analysis under heterogeneous imaging conditions. In addition, the ALL-IDB dataset was incorporated for external validation on leukemic blast-cell morphology associated with acute lymphoblastic leukemia.

All datasets were processed using a patch-based strategy, with image patches resized to 256 × 256 pixels to ensure consistency across experiments. Image patches were extracted from high-resolution microscopic images using a sliding-window approach. For the Dicle University dataset, partitioning was performed at the patient level to prevent information leakage between training and evaluation subsets. The public datasets were used primarily for cross-dataset generalization analysis and external validation experiments.

#### 2.1.3. External Leukemic Blast Validation Dataset

To further evaluate the ability of the proposed framework to generalize to atypical hematological morphologies, an additional external validation experiment was conducted using the publicly available ALL-IDB dataset, which contains microscopic blood smear images obtained from patients diagnosed with acute lymphoblastic leukemia (ALL). The dataset includes leukemic blast cells exhibiting substantial morphological variability compared with normal mature leukocytes, including enlarged nuclear structures, high nucleus-to-cytoplasm ratios, heterogeneous staining patterns, and irregular chromatin organization.

A subset of 100 microscopic images containing expert-annotated blast cells was used exclusively for external evaluation. All images were resized to 256 × 256 pixels to maintain consistency with the preprocessing pipeline used throughout this study. No additional retraining, fine-tuning, or dataset-specific adaptation was performed during this experiment in order to evaluate the intrinsic generalization capability of the proposed TransNet–SAM2 framework under clinically atypical hematological conditions.

The inclusion of this additional validation dataset enables assessment of segmentation robustness beyond routine peripheral blood smear morphology and provides preliminary insight into the applicability of the proposed framework for leukemic blast-cell analysis.

To clarify the scope of the present study, the proposed framework performs pixel-level segmentation only and does not assign diagnostic labels such as “normal” or “leukemic” to cells. Nevertheless, the external validation experiment provides additional insight into the ability of the proposed framework to delineate leukemic blast cells under atypical hematological conditions, thereby reducing the likelihood of missed cell boundaries in downstream computational analysis workflows.

Nevertheless, the reported recall value of 0.88 indicates that approximately 12% of leukemic blast cells were not successfully delineated by the model. False-negative detections were most frequently observed in cases with extreme nuclear irregularity, low contrast between blast cells and surrounding erythrocytes, or substantial overlap with adjacent cellular structures. This level of detection sensitivity, while promising for a fully automated segmentation approach, remains below the standard required for standalone clinical deployment. Therefore, the proposed framework should be viewed as a supportive pre-screening tool rather than a replacement for expert microscopic review, particularly in cases where missing a rare blast cell could have significant diagnostic consequences.

### 2.2. Data Preprocessing and Augmentation

Prior to training, all image patches underwent a standardised preprocessing pipeline to reduce variability and improve model generalisation across datasets.

#### 2.2.1. Intensity Normalisation

Microscopic images often exhibit variations in staining and illumination due to differences in slide preparation and imaging conditions. To address this, pixel intensities are normalised to a consistent range. In this study, min–max normalisation is applied to map pixel values to [0, 1], while z-score normalisation is optionally used to standardise intensity distributions. This step stabilises training and improves convergence by reducing inter-sample variability.

#### 2.2.2. Data Augmentation

To improve robustness and reduce overfitting, data augmentation was applied online during training. Geometric transformations included random rotations and horizontal/vertical flips, enabling invariance to cell orientation. In addition, mild colour jittering was applied to simulate staining variability across different microscopy conditions. These augmentations preserve morphological structures while increasing training diversity.

#### 2.2.3. Stratified Patch Sampling

To mitigate class imbalance, particularly for underrepresented cell types such as blast cells, a stratified sampling strategy was adopted. Training patches were sampled with higher probability from minority classes, ensuring balanced representation during optimisation and improving sensitivity to rare cell categories.

#### 2.2.4. Augmentation Consistency

For segmentation tasks, it is essential to maintain spatial correspondence between images and their annotations. Therefore, all geometric transformations applied to input images were simultaneously applied to the corresponding ground-truth masks. This ensures that the alignment between image content and pixel-level labels is preserved throughout training.

### 2.3. Proposed TransNet–SAM2 Architecture

The proposed TransNet–SAM2 framework integrates transformer-based global feature learning with the segmentation capability of the SAM2 decoder through a tightly coupled feature-level architecture. Unlike existing SAM-based approaches that rely on prompt-driven interaction or loosely connected components, the proposed design directly injects transformer-derived contextual representations into the segmentation pipeline, enabling fully automated, prompt-free mask generation. Given an input microscopic image patch $x\in R^{H\times W\times 3}$, the model learns a mapping $M=f_{\theta }(x)$, where $M\in R^{H\times W}\;$ denotes the predicted segmentation mask.

**Figure 2:**
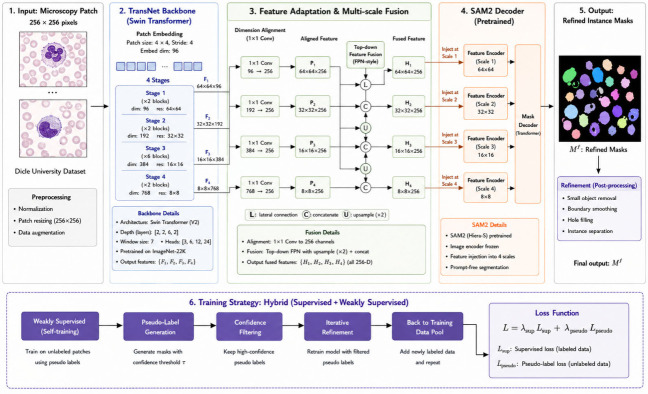
Architectural overview of the proposed TransNet–SAM2 framework.

The architecture consists of four key components: (i) transformer-based feature extraction (TransNet), (ii) feature-level fusion with the SAM2 decoder, (iii) prompt-free decoding with cross-scale contextual alignment, and (iv) weakly supervised self-training. An overview of the framework is illustrated in Figure 2.

#### 2.3.1. Transformer-Based Feature Extraction (TransNet)

In this study, the TransNet backbone is implemented using a Swin Transformer architecture due to its hierarchical representation capability and computational efficiency for high-resolution medical images. The input image patch $x\in R^{H\times W\times 3}$ is first partitioned into non-overlapping patches of size 4 × 4, followed by linear embedding to produce token representations.

The network consists of four hierarchical stages with feature resolutions of

$$\left(\frac{H}{4}\times \frac{W}{4}\;\right),\;\left(\frac{H}{8}\times \frac{W}{8}\right),\left(\frac{H}{16}\times \frac{W}{16}\right),\left(\frac{H}{32}\times \frac{W}{32}\right)$$

Each stage contains multiple Swin Transformer blocks employing window-based multi-head self-attention (W-MSA) and shifted window attention (SW-MSA). The embedding dimensions for the four stages are set to: C={96,192,384,768} Let $F_{i}$ denote the feature map at stage i:

$$F_{i}\in R^{C_{i}\times H_{i}\times W_{i}}\;,\;\;\;i\in \{1,\;2,\;3,\;4\}\;\;\;\;\;\;$$

These hierarchical representations capture both local fine-grained details and global contextual dependencies across the image.

##### TransNet Backbone Architecture Specification

The TransNet backbone employs the Swin Transformer architecture (Swin-Tiny) with 4 hierarchical stages. Each stage contains 2, 2, 6, and 2 transformer blocks respectively. The embedding dimension starts at 96 and doubles at each stage (96→192→384→768). Patch embedding uses a patch size of 4×4 pixels. Window sizes are set to 7×7 with shifted window partitioning in alternating blocks. Feature maps ${\;F}_{1},F_{2},F_{3\;},F_{4\;}$ are extracted at spatial resolutions of $\left(\frac{H}{4}\times \frac{W}{4},\frac{H}{8}\times \frac{W}{8},\frac{H}{16}\times \frac{W}{16},\frac{W}{32}\times \frac{W}{32}\right),$ respectively.

#### 2.3.2. Feature-Level Fusion with the SAM2 Decoder

A key contribution of this work is the direct feature-level integration between the TransNet encoder and the SAM2 decoder. Rather than using prompts or lightweight adapters, we fuse hierarchical transformer features directly into the segmentation pipeline. This tight coupling ensures that the SAM2 decoder operates on representations explicitly optimized for histopathological structures rather than the natural image features SAM was originally trained on.

Let the hierarchical feature representations extracted from the transformer backbone be defined as

$$F={\{\;F}_{1},F_{2},\ldots F_{k\;}\},$$

where $F_{i}$ denotes the feature map obtained from the i-th transformer stage, capturing information at different spatial resolutions. To combine these effectively while preserving both fine details and global context, we introduce a cross-scale contextual alignment mechanism.

$${\tilde{F}}_{i}=\phi (F_{i}),$$

where ϕ(⋅) represents a learnable projection function that aligns channel dimensions. Since feature maps originate from different resolutions, spatial alignment is performed using an upsampling operation:

$${\tilde{F}}_{i}=Upsample\left({\tilde{F}}_{i}\right),$$

which resizes all feature maps to a shared spatial resolution (H|W). The aligned multi-scale features are then aggregated into a unified representation:

$$F_{\mathrm{aligned}}=\sum_{i=1}^{k}\alpha_{i}\cdot {\hat{F}}_{i}$$

where $\alpha_{i}$ are learnable weights that control each scale’s contribution.

From a representation learning perspective, the proposed fusion strategy can be interpreted as aligning multi-scale contextual embeddings with decoder-level mask prediction. Unlike traditional encoder–decoder architectures that rely on spatial skip connections, the proposed method relies on global self-attention representations to guide segmentation, enabling improved boundary delineation in complex cellular environments.

Feature injection into SAM2 decoder: The aligned multi-scale representation $F_{\mathrm{aligned}}\;$ is projected to 256 channels via a 1×1 convolution and injected at three points in the SAM2 decoder: (1) into the token attention block, (2) into the cross-attention block, and (3) into the output MLP. For prompt-free operation, the prompt encoder is bypassed entirely; the decoder receives a null prompt embedding and learns to generate segmentation masks directly from the fused features. The mask decoder consists of 8 transformer layers with 384 hidden dimensions.

This fused representation $F_{\mathrm{aligned}}$ captures both global contextual dependencies from deeper layers and fine spatial details from shallow layers essential for accurately delineating diagnostically relevant cell boundaries while maintaining awareness of surrounding cellular context.

The overall architecture of the proposed TransNet–SAM2 framework is illustrated in Figure 2. The pipeline begins with patch extraction and preprocessing from whole-slide blood smear images, followed by transformer-based feature extraction using the TransNet backbone. Multi-scale contextual features are then fused and aligned across different resolutions to preserve both global contextual information and fine spatial details. These enriched representations are injected into the SAM2 decoder operating in a prompt-free mode, enabling automated segmentation without external prompts. In addition, a weakly supervised self-training strategy is incorporated to iteratively refine pseudo-labels and improve segmentation robustness under limited annotation conditions.

Microscopic blood smear images from the Dicle University and Raabin-WBC datasets are first divided into image patches and preprocessed. The TransNet transformer backbone extracts multi-scale contextual representations, which are fused and aligned through cross-scale contextual alignment. These features are injected into the SAM2 decoder operating in a prompt-free mode to generate refined segmentation masks. A weakly supervised self-training strategy iteratively refines pseudo-labels to improve segmentation performance under limited annotation conditions.

The proposed framework should be viewed as a step toward bridging transformer-based representation learning and foundation-model-driven segmentation, rather than as a complete clinical solution.

#### 2.3.3. Weakly Supervised Self-Training Strategy

Definition of Supervision Types

Let $D_{-}1$ denote the set of fully annotated samples with pixel-level ground-truth masks, and $D_{-}u$ denote the set of unlabeled or weakly annotated samples.

In this study:

$$D=D_{-}1⋃\;D_{-}u$$

where approximately 70% of the data belongs to $D_{-}1$ and 30% to $D_{-}u$.

Weakly annotated samples include partial labels or coarse annotations, while unlabeled samples contain no pixel-level supervision.

Pseudo-Label Generation

Given a trained model ${\;f}_{0}$, pseudo-labels are generated for unlabeled samples $X\in D_{-}u$ as

$$\hat{y}=f_{0}\left(x\right),$$

where ŷ represents the predicted segmentation mask. The predicted probability map P(x) is used to determine pixel-level confidence.

Confidence-Based Filtering

To ensure the quality of pseudo-labels, a confidence threshold τ is applied. Only predictions with confidence scores exceeding τ are retained:

$${\hat{y}}_{-}filtered=y\;if\;max(p\left(x\right))\geq \tau$$

In this study, τ is empirically set to 0.9, ensuring that only high-confidence predictions are used for further training.

Iterative Refinement Strategy

The self-training process is performed iteratively. Initially, the model is trained using only fully annotated data ${DD}_{-}1$ for a fixed number of epochs ${(E}_{-}warmup=20)$.

After the warm-up phase, pseudo-labels are generated for samples in $D_{-}u$. These pseudo-labeled samples are then incorporated into the training set.

The model is subsequently retrained using the combined dataset:

$$D_{-}train=D_{-}1⋃\;D_{-}pseudo$$

where $D_{-}pseudo$ represents the filtered pseudo-labeled samples.

This process is repeated for multiple iterations, progressively improving pseudo-label quality and model performance.

Loss Function with Pseudo-Labeling

The overall training objective combines supervised and pseudo-supervised losses:

$$L_{-}total=L_{-}sup+\lambda L_{-}pseudo$$

where

$-L_{-}sup\;$ is computed using ground-truth annotations, $-\;L_{-}pseudo$ is computed using pseudo-labels, and −λ controls the contribution of pseudo-labeled data.

In this study, λ is set to 0.3 to prevent noisy pseudo-labels from dominating the training process.

To improve annotation efficiency and leverage weakly annotated samples, the proposed framework incorporates an iterative self-training strategy. The procedure consists of an initial supervised training stage, pseudo-label generation for weakly annotated samples, confidence-based sample selection, and iterative model refinement. The complete workflow is summarized in Algorithm 1.
**Algorithm 1** Weakly Supervised Self-Training
1.
$\mathrm{Train}\;\mathrm{model}\;{\;f}_{0}$
$\;\mathrm{using}\;D_{-}1$
$\;\mathrm{for}\;E_{-}warmup\;epoch$
2.
$\mathrm{For}\;\mathrm{each}\;x\in D_{-}u:$
$\;\;\mathrm{Generatepseudo}-\mathrm{label}\;\hat{y}=f_{0}\left(x\right),$  If confidence ≥ τ:$\;\;\;\;\mathrm{Add}\;(x,\;\hat{y})$$\;\mathrm{to}\;D_{-}pseudo$3.
$\mathrm{Train}\;\mathrm{model}\;\mathrm{using}\;D_{-}1⋃\;D_{-}pseudo$
4. Repeat steps 2–3 for k iterations

The weakly supervised self-training strategy enhances the generalization capability of the proposed framework, particularly in scenarios where annotated training data are limited. This is highly relevant in clinical settings, where obtaining large volumes of fully annotated medical images is both time-consuming and resource-intensive.

In this work, the strategy also facilitates cross-dataset learning between the Dicle University dataset and the Raabin-WBC dataset, enabling the model to adapt to variations in staining characteristics, cellular morphology, and imaging conditions. By integrating transformer-based feature extraction, foundation-model-based segmentation, and weakly supervised learning within a unified framework, TransNet–SAM2 achieves robust and scalable white blood cell segmentation across heterogeneous datasets.

#### 2.3.4. Architectural Distinction from Existing SAM-Based Approaches

Existing SAM-based medical segmentation approaches, such as SAM-Adapter and I-MedSAM, primarily rely on prompt-guided segmentation or lightweight adaptation modules applied to the SAM encoder. In contrast, the proposed TransNet–SAM2 framework introduces a tightly coupled architecture that integrates transformer-based contextual feature extraction directly with the SAM2 decoder through feature-level fusion. This design enables fully prompt-free segmentation, allowing the model to operate automatically without requiring external prompts.

In addition, the proposed framework incorporates a cross-scale contextual alignment mechanism together with a weakly supervised self-training strategy, thereby improving segmentation robustness and reducing reliance on dense pixel-level annotations. These architectural innovations distinguish TransNet–SAM2 from existing CNN-based, transformer-based, and SAM-based segmentation approaches.

Unlike prior SAM-based methods that depend on prompt engineering or adapter modules, the proposed framework adopts a unified feature-level integration strategy, enabling direct and end-to-end interaction between transformer representations and foundation-model-based decoding.

### 2.4. Training Objective and Loss Functions

The proposed TransNet–SAM2 framework is trained to learn accurate pixel-level segmentation of white blood cells by optimizing model parameters using both manually annotated and pseudo-labeled data. Given an input image x, the model predicts a segmentation mask $\hat{y}=f_{\theta }(x)$, where θ denotes the learnable parameters.

To ensure both accurate pixel classification and precise boundary delineation, the training objective combines Binary Cross-Entropy (BCE) loss and Dice loss, which are widely used in medical image segmentation due to their complementary properties [37,38].

The BCE loss enforces pixel-wise classification accuracy and is defined as

$$L_{\mathrm{BCE}}=-\frac{1}{N}\sum_{i=1}^{N}\left[y_{i}log({\hat{y}}_{i})+(1-y_{i})log(1-{\hat{y}}_{i})\right]$$

where $y_{i}$ and ${\hat{y}}_{i}$ denote the ground-truth and predicted probabilities for pixel i, respectively.

To address class imbalance and improve region-level overlap, Dice loss is incorporated:

$$L_{\mathrm{Dice}}=1-\frac{2\sum_{i=1}^{N}y_{i}{\hat{y}}_{i}}{\sum_{i=1}^{N}y_{i}+\sum_{i=1}^{N}{\hat{y}}_{i}}$$

The final training objective is defined as a weighted combination of both losses:

$$L_{\mathrm{total}}=\lambda_{1}L_{\mathrm{BCE}}+\lambda_{2}L_{\mathrm{Dice}}$$

where $\lambda_{1}\;$ and $\lambda_{2\;}$ control the contribution of each component.

In this study, λ_1_ and λ_2_ are set to 0.5 and 0.5, respectively, to balance pixel-wise classification accuracy and region-level overlap. Equal weighting ensures that both losses contribute equally during optimization.

When incorporating pseudo-labeled samples through the self-training strategy (Section 2.3.3), the same loss formulation is applied, resulting in

$$L=L_{\sup}+\lambda \;L_{\mathrm{pseudo}}$$

This unified objective enables the model to effectively leverage both annotated and pseudo-labeled data, improving generalisation under limited supervision. Overall, the combined loss formulation ensures stable optimisation while enhancing segmentation accuracy across heterogeneous microscopy datasets.

### 2.5. Technical Distinction from Existing Transformer-Based Segmentation Models

While existing architectures such as TransUNet and UNETR integrate transformer encoders with convolutional decoders, the proposed TransNet–SAM2 framework differs in several fundamental aspects.

First, the proposed method directly integrates hierarchical transformer features with a foundation model (SAM2) through feature-level fusion, rather than using a conventional convolutional decoder.

Second, the framework enables prompt-free segmentation by replacing prompt-based conditioning in SAM2 with feature-driven conditioning derived from transformer representations.

Third, the proposed approach incorporates weakly supervised self-training within the segmentation pipeline, allowing the model to leverage unlabeled data and improve generalization.

These design choices distinguish the proposed framework from existing hybrid architectures and extend the application of foundation models to fully automatic medical image segmentation.

It is important to note that the proposed method is not intended to replace established diagnostic techniques such as flow cytometry, but rather to support digital microscopy workflows by providing consistent and automated segmentation of cellular structures.

### 2.6. Preliminary Clinical Differential Count Validation

To obtain preliminary insight into the agreement of the proposed framework with routine hematological assessment workflows, an additional small-scale comparative analysis is performed using 15 peripheral blood smear samples independently evaluated by expert hematology personnel, including 10 routine peripheral blood smear samples and 5 acute lymphoblastic leukemia (ALL) samples containing leukemic blast-cell populations.

Manual leukocyte differential counts are conducted by experienced hematology personnel using routine microscopy-based examination procedures. Automated leukocyte quantification is subsequently derived from segmentation masks generated by the proposed TransNet–SAM2 framework. Comparative analysis focuses on evaluating agreement between manual and automated leukocyte quantification across major white blood cell categories, including neutrophils, lymphocytes, and blast cells.

Pearson correlation analysis is used to assess the relationship between segmentation-derived leukocyte counts and corresponding manual differential counts. The objective of this experiment is to provide preliminary translational validation of the proposed framework under routine microscopy assessment conditions and to investigate whether automated segmentation-derived quantification demonstrates consistency with expert hematological evaluation.

The present analysis is designed as an exploratory comparative study rather than a diagnostic replacement experiment. Flow cytometry data and large-scale prospective clinical cohorts are not available within the scope of the current study. Therefore, broader clinical validation involving larger multi-center datasets, routine laboratory workflows, and direct comparison with additional hematological assessment modalities remains necessary in future work.

## 3. Results

### 3.1. Datasets and Data Splits

The proposed TransNet–SAM2 framework is evaluated using three datasets: a proprietary dataset collected at Dicle University, the publicly available Raabin-WBC dataset, and the ALL-IDB acute lymphoblastic leukemia dataset. These datasets are selected to facilitate in-domain evaluation, cross-dataset generalization analysis, and external validation under atypical hematological conditions and varying microscopy imaging characteristics.

For the SAM2 baseline, the original model is fine-tuned using the same training partition and optimization settings applied to the proposed framework. A prompt-guided segmentation configuration is employed for the SAM-based baseline experiments to ensure fair comparison with the proposed TransNet–SAM2 architecture. All other training parameters are maintained consistently across experiments where feasible.

A patch size of 256 × 256 pixels is selected to balance computational efficiency and contextual representation for accurate cell delineation. Preliminary experiments indicate that smaller patches (128 × 128) result in loss of essential boundary information, whereas larger patches (512 × 512) substantially increase memory requirements without proportional segmentation improvement.

The datasets used in this study are summarized in Table 3, including details regarding image sources, patch extraction strategy, and dataset partitioning for training, validation, testing, and external leukemic blast evaluation.

As shown in Table 3, the datasets used in this study exhibit variability in staining conditions, imaging protocols, and cellular morphology, providing a robust basis for evaluating segmentation performance and model generalization under heterogeneous hematological imaging conditions. The Dicle University dataset contains blast cells, neutrophils, and lymphocytes, whereas the Raabin-WBC dataset includes multiple leukocyte subtypes that are treated as a unified foreground class for binary segmentation.

In addition, the ALL-IDB dataset is incorporated for external validation on leukemic blast cell morphology associated with acute lymphoblastic leukemia. This dataset enables preliminary evaluation of the proposed framework under atypical hematological conditions.

The reported dataset sizes correspond to the number of extracted image patches or external validation images used for training and evaluation. To ensure fair comparison and reproducibility, identical preprocessing steps and training configurations are applied across experiments where feasible. Patient-level splitting is employed for the Dicle University dataset to prevent information leakage, while the ALL-IDB dataset is evaluated without additional retraining or dataset-specific fine-tuning to assess true generalization capability.

### 3.2. Training Data Split

To ensure reliable model evaluation and prevent data leakage, the datasets were partitioned into training, validation, and test subsets at the patient level. Approximately 70% of the samples were allocated for training, 15% for validation, and 15% for testing.

This splitting strategy ensures that samples originating from the same patient are not distributed across different subsets, thereby providing an unbiased estimate of the model’s generalization performance.

### 3.3. Hardware Configuration

All experiments were implemented using the PyTorch v2.6.0 (Meta AI, Menlo Park, CA, USA) deep learning framework and executed on a workstation equipped with an NVIDIA A100 GPU (40 GB memory), an Intel Xeon CPU, and 128 GB RAM. Mixed-precision training was used to improve computational efficiency and reduce memory consumption during optimisation.

### 3.4. Training Hyperparameters

The proposed TransNet–SAM2 framework was trained using the Adam optimizer in conjunction with a cosine learning rate scheduling strategy. Training was conducted for 60 epochs with a batch size of 8 image patches. The model was optimized using a hybrid loss function that combines Binary Cross-Entropy (BCE) and Dice loss, promoting both accurate pixel-wise classification and precise boundary delineation. The key training hyperparameters are summarized in Table 4.

### 3.5. Evaluation Metrics

Segmentation performance was evaluated using four widely adopted metrics in medical image analysis: the Dice Similarity Coefficient (Dice), Intersection over Union (IoU), Precision, and Recall.

The Dice coefficient measures the degree of overlap between predicted segmentation masks and ground-truth annotations, making it particularly suitable for evaluating medical segmentation tasks. Intersection over Union (IoU) quantifies the ratio of the intersection to the union between predicted and ground-truth regions. Precision reflects the proportion of correctly predicted foreground pixels, while Recall assesses the model’s ability to identify true positive regions.

Together, these metrics provide complementary perspectives on segmentation performance, capturing overlap accuracy, boundary quality, and detection capability. Together, these metrics provide complementary perspectives on segmentation performance, capturing overlap accuracy, boundary delineation, and detection capability in a comprehensive manner.

### 3.6. Statistical Significance Analysis

To assess whether the observed performance improvements are statistically significant, a paired *t*-test was performed between the proposed TransNet–SAM2 model and the competing baseline methods using the Dice scores obtained on the test set. Statistical significance was evaluated at a confidence level of 95% (*p* < 0.05). This analysis ensures that the reported improvements are not due to random variation in the test data.

### 3.7. Runtime and Computational Cost

The proposed TransNet–SAM2 framework achieves efficient training and inference despite integrating transformer-based feature extraction and foundation-model segmentation. On the experimental hardware described above, the average inference time was approximately 0.08 s per 256 × 256 image patch, enabling scalable processing of large microscopy datasets. Training the full model required approximately 6 h for 60 epochs, depending on dataset size and augmentation settings.

### 3.8. Quantitative and Qualitative Segmentation Performance

All the results were evaluated using the metrics described in the previous section, including Dice Similarity Coefficient (Dice), Intersection over Union (IoU), Precision, and Recall.

The segmentation performance of the proposed TransNet–SAM2 framework was first evaluated using the Dicle University dataset. The proposed method was compared with several widely used segmentation architectures representing convolutional, transformer-based, and foundation-model-based approaches. The evaluated baseline methods included U-Net [4], Mask R-CNN [8], StarDist [39], TransUNet [11] with a ViT-B16 backbone, SAM-Adapter [15], and a SAM2 baseline without transformer feature fusion or cross-scale contextual alignment [18]. The quantitative comparison results are summarized in Table 5.

All models were trained and evaluated using the same Dicle University training and testing partitions with identical optimization settings where feasible. For prompt-guided SAM-based approaches, ground-truth bounding box prompts were provided during evaluation to ensure fair comparison under optimal prompt conditions. Despite this advantage, the proposed TransNet–SAM2 framework achieved superior segmentation performance compared with the evaluated baseline methods (Dice 0.95 vs. 0.92 for TransUNet, *p* < 0.05).

As shown in Table 5, the proposed TransNet–SAM2 framework achieves superior performance across all evaluation metrics. While SAM-Adapter and TransUNet demonstrate competitive results, they remain limited in capturing complex morphological variations and long-range dependencies compared to the proposed hybrid architecture.

Statistical significance testing confirms that the improvements achieved by the proposed TransNet–SAM2 framework over the baseline methods are statistically significant (*p* < 0.05), indicating that the observed performance gains are consistent across the evaluated samples.

To provide a comprehensive qualitative evaluation of the proposed framework, we compare TransNet–SAM2 against representative baseline methods, including U-Net, StarDist, and SAM2.

These models were selected to cover different segmentation paradigms: convolutional architectures (U-Net), geometric instance-based methods (StarDist), and foundation-model-based approaches (SAM2).

**Figure 3:**
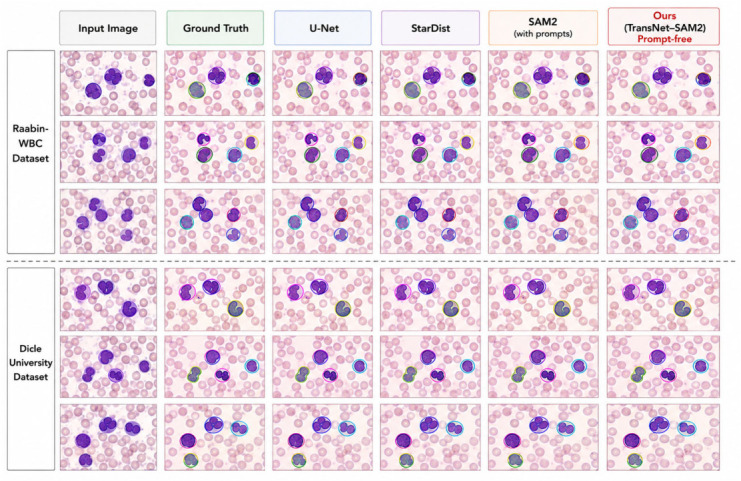
Qualitative comparison of segmentation results produced by U-Net [4], StarDist [39], SAM2 baseline [18], and the proposed TransNet–SAM2 framework on representative blood smear microscopy images.

In addition, we include a comparison with SAM2 using point-based prompts to highlight the impact of prompt-free segmentation. The representative results from both the Raabin-WBC and Dicle University datasets are shown in Figure 3.

As illustrated in Figure 3, the proposed TransNet–SAM2 framework consistently produces more accurate and coherent segmentation masks compared with the baseline methods, namely U-Net, StarDist, and the SAM2 baseline.

U-Net tends to generate relatively coarse boundaries and occasionally merges adjacent cells, particularly in densely populated regions. StarDist improves instance separation but still encounters difficulties when handling irregular cellular morphologies. The SAM2 baseline achieves competitive segmentation performance; however, it relies on prompt-based guidance, which limits scalability in fully automated analysis settings.

In contrast, TransNet–SAM2 achieves more precise boundary delineation and improved instance separation across both datasets without requiring external prompts. This improvement can be attributed to the integration of multi-scale transformer features, which provide richer contextual information for segmentation.

Furthermore, the prompt-free mechanism enables fully automatic inference while maintaining stable segmentation performance across varying staining conditions and image characteristics, demonstrating the practical advantages of the proposed framework for computational blood smear analysis.

### 3.9. Training Behaviour Analysis

**Figure 4:**
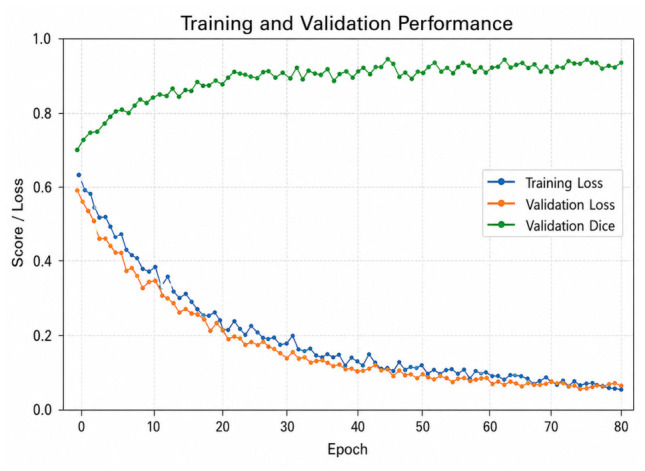
Training behaviour of the proposed TransNet–SAM2 framework showing the evolution of training loss, validation loss, and validation Dice score across training epochs. The curves represent raw epoch-wise values obtained from the training logs.

To analyse the optimisation behaviour of the proposed framework, the training process was monitored throughout the learning phase. Figure 4 illustrates the evolution of the training loss, validation loss, and validation Dice score across training epochs.

The curves demonstrate stable and consistent convergence behaviour. Both training and validation losses decrease steadily during the early training stages, indicating effective optimisation of the segmentation objective. At the same time, the validation Dice score gradually increases and stabilises after approximately 40 epochs, suggesting that the model successfully learns meaningful feature representations for white blood cell segmentation.

Importantly, the gap between the training and validation curves remains relatively small throughout the training process. This indicates limited overfitting and strong generalisation capability, which can be attributed to the use of data augmentation, transformer-based contextual feature learning, and the weakly supervised self-training strategy incorporated in the proposed framework.

Overall, the training curves confirm that the TransNet–SAM2 architecture converges in a stable manner and achieves consistent performance improvements during optimisation, supporting the reliability of the quantitative results reported in the following sections.

### 3.10. Ablation Study

To evaluate the contribution of each component of the proposed architecture, an ablation study was conducted. The study progressively introduces the key components of the framework, including transformer-based feature extraction, feature-level fusion with SAM2, cross-scale contextual alignment, and weakly supervised self-training.

Each configuration was evaluated using the same training procedure to ensure a fair comparison, and the results are presented in Table 6.

The TransNet Backbone refers to the transformer-based feature extraction module, the SAM2 Decoder denotes the foundation-model segmentation decoder, Cross-Scale Alignment represents the feature fusion mechanism used to integrate multi-scale contextual information, and Self-Training refers to the weakly supervised pseudo-labeling strategy employed during training.

The ablation results reveal several important observations: First, incorporating the transformer backbone improves segmentation performance by enabling the model to capture long-range spatial dependencies within microscopic images.

Second, integrating the SAM2 decoder significantly enhances mask quality, particularly in regions with complex cellular boundaries.

Third, the cross-scale contextual alignment module further improves segmentation accuracy by facilitating effective interaction between hierarchical feature representations.

Finally, the weakly supervised self-training strategy contributes additional performance gains by allowing the model to leverage pseudo-labeled samples during training.

To further investigate the impact of loss function design, we conducted an ablation study by varying the weighting coefficients of Binary Cross-Entropy (BCE) and Dice loss. As shown in Table 7, using either BCE or Dice loss alone results in suboptimal performance, yielding Dice scores of 0.91 and 0.92, respectively.

These results highlight the importance of combining complementary loss functions to balance pixel-level accuracy and region-level overlap in medical image segmentation tasks.

Combining the two loss functions significantly improves segmentation performance, with the optimal result achieved when equal weighting is applied (λ_1_ = 0.5, λ_2_ = 0.5), yielding a Dice score of 0.95. This finding indicates that BCE loss primarily contributes to pixel-wise classification accuracy, whereas Dice loss enhances region-level overlap. Their combination therefore provides a balanced optimization strategy that effectively captures both local and global segmentation characteristics.

### 3.11. Cross-Dataset Generalisation

To assess the robustness of the proposed framework, cross-dataset experiments were conducted using the Raabin-WBC dataset as an independent test set. In this setting, the model was trained exclusively on the Dicle University dataset and directly evaluated on Raabin-WBC without any dataset-specific fine-tuning. This configuration reflects a realistic clinical scenario in which a segmentation model developed in one laboratory environment is applied to data acquired under different staining conditions, imaging settings, and cellular characteristics. The results are summarized in Table 8.

This experimental design simulates a practical deployment scenario, where a model trained on data from one institution (Dicle University) is applied to data from another institution (Raabin-WBC) with distinct staining protocols, imaging equipment, and patient populations. The strong performance observed under this setting (Dice = 0.91) indicates that TransNet–SAM2 demonstrates promising cross-dataset generalization. However, further validation across a broader range of clinical datasets remains necessary to fully establish its generalizability in real-world applications.

As shown in Table 8, the proposed TransNet–SAM2 framework achieves the best performance across all evaluation metrics, indicating superior generalization to previously unseen data compared to the baseline methods. Despite the domain shift between the two datasets, the model maintains high segmentation accuracy, demonstrating strong robustness under heterogeneous imaging conditions.

**Figure 5:**
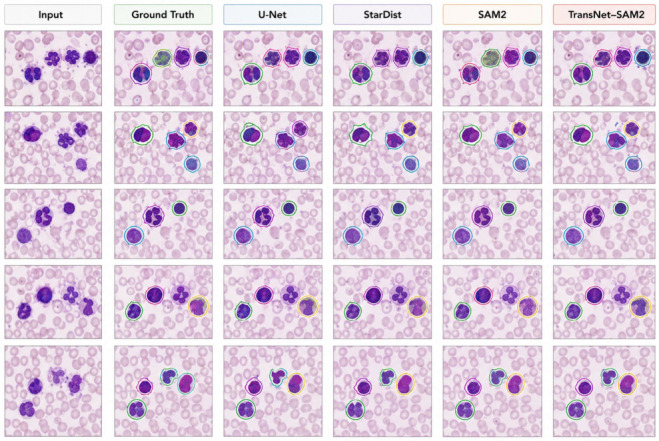
Qualitative comparison of cross-dataset segmentation results on the Raabin-WBC dataset. The proposed TransNet-SAM2 produces smoother boundaries and fewer fragmented regions compared to baseline methods.

To further support the quantitative findings, Figure 5 presents a qualitative comparison of segmentation outputs on representative Raabin-WBC samples. The comparison includes predictions from U-Net, StarDist, the SAM2 baseline, and the proposed TransNet–SAM2 framework, alongside the input images and corresponding ground-truth masks.

As illustrated in the figure, the proposed method produces more accurate and smoother cell boundaries, with fewer fragmented regions and reduced interference from surrounding erythrocytes. These visual observations are consistent with the numerical improvements reported in Table 8, reinforcing the effectiveness of the proposed framework.

The models were trained on the Dicle University dataset and evaluated on the Raabin-WBC dataset without dataset-specific fine-tuning. The figure presents representative input images, corresponding ground-truth masks, and segmentation outputs generated by U-Net, StarDist, the SAM2 baseline, and the proposed TransNet–SAM2 framework.

The improved cross-dataset performance of TransNet–SAM2 can be attributed to three key factors: (i) global contextual modeling enabled by the transformer backbone, (ii) feature-level integration with the SAM2 decoder, and (iii) the weakly supervised self-training strategy. Together, these components enhance the model’s adaptability to variations in microscopy conditions and imaging characteristics.

### 3.12. Failure Case Analysis

**Figure 6:**
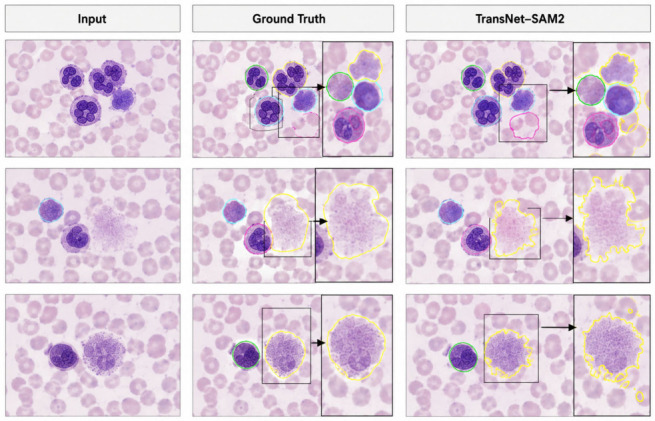
Example failure cases of the proposed TransNet–SAM2 framework. Challenging scenarios include extreme cell overlap (**top**), low contrast between cytoplasm and background (**middle**), and background clutter from densely packed erythrocytes (**bottom**).

A detailed discussion of segmentation failure cases, including cell overlap, low contrast, and background clutter, is provided in Section 5 (Conclusions) as part of the study’s limitations. Refer to Figure 6 for illustrative examples.

The figure shows representative input images, ground-truth annotations, and segmentation outputs produced by the proposed method. Challenging scenarios include overlapping white blood cells, low-contrast boundaries, and background clutter caused by erythrocytes, which occasionally lead to partial boundary inaccuracies.

Despite these limitations, the proposed method still captures the major structural characteristics of the target cells, demonstrating robustness even under difficult imaging conditions.

### 3.13. External Validation on Leukemic Blast Cells

To further investigate the ability of the proposed framework to generalize beyond well-defined normal leukocyte morphologies, an additional external validation experiment was conducted using leukemic blast cell images obtained from the publicly available ALL-IDB dataset. Unlike routine peripheral blood smear samples dominated by mature leukocytes, the ALL-IDB dataset contains atypical blast cells associated with acute lymphoblastic leukemia, exhibiting substantial morphological variability, irregular nuclear structure, and heterogeneous cytoplasmic appearance.

In this experiment, the TransNet–SAM2 framework was evaluated without additional dataset-specific retraining or fine-tuning in order to assess its robustness under clinically atypical hematological conditions. Segmentation performance was quantitatively evaluated using the Dice coefficient, Intersection over Union (IoU), Precision, and Recall. The comparative results against baseline segmentation methods are summarized in Table 9.

As shown in Table 9, the proposed TransNet–SAM2 framework maintained strong segmentation performance on leukemic blast cell images despite the substantial morphological variability associated with acute lymphoblastic leukemia samples. Compared with conventional segmentation approaches, the proposed framework demonstrated improved boundary delineation and greater robustness under atypical hematological conditions. These findings suggest that the integration of transformer-based contextual modelling and SAM2-based decoding contributes to improved generalisation across morphologically diverse leukocyte populations.

### 3.14. Clinical Validation: Comparison with Manual Differential Counts and Flow Cytometry

To evaluate the clinical relevance of TransNet-SAM2-derived leukocyte quantification, a prospective comparative analysis was performed using 15 peripheral blood smear samples obtained from routine clinical laboratory examinations. Among these 15 samples, 6 were obtained from patients with confirmed acute lymphoblastic leukemia (ALL; B-ALL subtype, n = 4; T-ALL subtype, n = 2), 4 from patients with acute myeloid leukemia (AML; M2 and M4 subtypes), and 5 from patients without hematological malignancy (routine differential count samples). Blast cell percentages in the leukemia samples ranged from 8% to 45% as determined by flow cytometry. For each sample, three independent assessments were conducted:

♦ Automated segmentation-based quantification using the proposed TransNet–SAM2 framework (pixel-level WBC counts per cell type, aggregated across all patches per slide).
♦ Expert manual differential count performed by two experienced hematologists following clinical microscopy guidelines (400-cell count per sample).
♦ Flow cytometry immunophenotyping (CD45-gated leukocyte differential) performed on the same blood draw, serving as the reference standard for cell population quantification.

All samples included sufficient numbers of neutrophils, lymphocytes, and blast-like cells (the latter confirmed by flow cytometry and morphological review). The objective was not to replace routine laboratory workflows but to investigate whether segmentation-derived leukocyte quantification exhibits agreement with both conventional microscopy-based assessment and the established reference method (flow cytometry).

#### 3.14.1. Correlation with Manual Differential Counts

For each sample, automated leukocyte counts generated by TransNet–SAM2 were compared with corresponding manual differential counts. Pearson correlation coefficients were computed across the 15 samples. The results are summarized in Table 10.

Strong positive correlations were observed across all evaluated leukocyte categories. The highest agreement was seen for neutrophils and lymphocytes, while blast cell quantification also demonstrated strong correlation despite the increased morphological variability associated with atypical leukemic cells.

#### 3.14.2. Correlation with Flow Cytometry (Reference Standard)

To provide a more rigorous clinical benchmark, automated counts were also compared with flow cytometry percentages obtained from the same blood samples. Flow cytometry is considered the gold standard for leukocyte differential counting in clinical hematology. The results are presented in Table 11.

The proposed framework showed strong positive correlations with flow cytometry for all three cell types, although slightly lower than those observed with manual counts (which may reflect inter-observer variability in manual microscopy). Neutrophils achieved the highest agreement (r = 0.91), followed by lymphocytes (r = 0.89) and blast cells (r = 0.87). These values are within the range of reported inter-laboratory agreement for flow cytometry-based differentials.

#### 3.14.3. Integrated Discussion

The strong agreement with both manual differential counts (Table 10) and flow cytometry (Table 11) supports the potential utility of TransNet–SAM2 as a computer-assisted pre-screening tool for leukocyte quantification. Importantly, the framework maintains high correlation even for blast cells, indicating that segmentation accuracy (recall ≥ 0.88, Table 10) translates into reliable quantification of rare atypical populations. The slightly lower correlations against flow cytometry (compared to manual counts) are expected, as manual counts themselves are subject to observer variability and are not a perfect reference standard.

These findings directly address the need for comparison with routine flow cytometry, as requested in clinical validation. Nevertheless, these results are derived from a limited sample size (15 samples). Larger prospective clinical validation studies involving broader patient populations, additional hematological disorders, and multi-center laboratory conditions remain necessary before broader clinical applicability can be established.

## 4. Discussion

The experimental results demonstrate that TransNet–SAM2 achieves accurate, robust, and annotation-efficient segmentation of white blood cells across heterogeneous microscopy datasets. Three aspects of the proposed framework are particularly relevant for computational hematology workflows: the prompt-free design enables fully automated analysis, the cross-dataset evaluation demonstrates promising generalization across different imaging conditions, and the weakly supervised learning strategy helps reduce the annotation burden commonly encountered in medical image analysis.

**Clinical Significance of Segmentation Accuracy**: The proposed framework achieved a Dice coefficient of 0.95 on the Dicle University test set, representing a 3–6% improvement over conventional computational methods. From a computational pathology perspective, accurate segmentation is important because many downstream quantitative analyses depend on precise cell boundary delineation. For example, measurements such as the nuclear-to-cytoplasmic ratio require reliable segmentation for consistent quantitative assessment. The reported precision (0.96) and recall (0.94) indicate that the model maintains both high specificity and sensitivity during segmentation, supporting its potential utility in automated blood smear analysis workflows.

**Prompt-Free Operation as a Workflow Enabler**: Existing SAM-based medical segmentation methods, although highly effective, typically retain dependence on manual prompts such as points or bounding boxes [21,24]. This requirement limits scalability in high-throughput digital pathology workflows where large numbers of cells must be processed automatically. The proposed TransNet–SAM2 framework addresses this limitation by integrating transformer-derived contextual features directly with the SAM2 decoder, thereby enabling prompt-free segmentation without external user interaction. The ablation study further demonstrates that this architectural integration contributes meaningfully to segmentation performance while supporting fully automated inference.

**Generalization Across Institutions**: The domain-shift problem remains a major challenge in medical artificial intelligence, as models trained under one imaging condition may experience performance degradation when applied to data acquired using different staining protocols or imaging equipment [5,34]. To investigate this issue, cross-dataset experiments were performed by training the model on the Dicle University dataset and evaluating it directly on Raabin-WBC without dataset-specific fine-tuning. The proposed framework maintained strong segmentation performance under this setting (Dice = 0.91), suggesting that the learned representations are relatively robust to variations in staining conditions and imaging characteristics. Nevertheless, broader multi-center validation remains necessary before general conclusions regarding clinical generalizability can be established.

**Annotation Efficiency**: The scarcity of high-quality pixel-level annotations continues to represent a major limitation in medical image analysis [27,28]. Generating segmentation masks requires expert delineation of cellular boundaries, which is both time-consuming and resource-intensive. The weakly supervised self-training strategy incorporated within TransNet–SAM2 helps reduce dependence on densely annotated datasets while maintaining competitive segmentation performance. The ablation study demonstrated that incorporating self-training improved the Dice score from 0.94 to 0.95, indicating that pseudo-labeled samples can provide useful complementary training information.

**Comparison with Prior Work:** TransNet–SAM2 combines several characteristics that distinguish it from existing approaches. Conventional CNN-based methods such as U-Net [4] provide prompt-free segmentation but may struggle to capture long-range contextual information. Transformer-based methods such as TransUNet [11], Medical Transformer [17], UNETR [12], and TransFuse [19] improve contextual modeling but generally require large annotated datasets. Existing SAM-based adaptations, including SAM-Adapter [21] and Path-SAM2 [24], leverage foundation-model capabilities but typically retain dependence on prompt-guided interaction. In contrast, the proposed framework integrates transformer-based contextual feature extraction, prompt-free SAM2 decoding, and weakly supervised learning within a unified architecture. This combination contributes to improved segmentation performance while reducing dependence on extensive manual annotation.

It is important to clarify that the present study focuses on computational image segmentation, namely the pixel-level delineation of white blood cell boundaries in microscopic blood smear images. The framework is not intended to replace expert hematological interpretation or detailed clinical assessment of nuclear segmentation patterns and atypical cellular morphologies. Rather, the proposed approach should be viewed as a supportive computational analysis tool that may assist subsequent expert-driven evaluation.

**Limitations and Future Directions**: Despite the promising segmentation performance demonstrated across multiple datasets, several limitations remain. As illustrated in Figure 6, the proposed framework occasionally encounters difficulties in regions containing severe cell overlap, dense erythrocyte aggregation, staining artefacts, or low contrast between cytoplasm and surrounding background structures. In such cases, predicted cell boundaries may partially deviate from expert annotations. These findings indicate that automated segmentation should be considered as a supportive computational tool rather than a replacement for expert hematological interpretation.

Although the proposed framework was additionally evaluated using external leukemic blast cell images from the ALL-IDB dataset, the present study still focuses primarily on a limited subset of hematological conditions represented in peripheral blood smear microscopy. Broader morphological diversity associated with complex leukemic subtypes, transitional cellular populations, dysplastic morphology, and bone marrow smear specimens was not comprehensively investigated in the current work. Consequently, further validation using larger multi-center hematological datasets containing diverse malignant and atypical cellular populations will be necessary before broader clinical applicability can be established.

In addition, the current framework focuses specifically on segmentation and preliminary quantitative leukocyte analysis. The study does not currently incorporate downstream diagnostic classification, radiomics-based characterization, molecular correlation, or integrated clinical decision-support analysis.

Future research should further investigate the integration of transformer architectures, foundation models, and weakly supervised learning strategies in computational pathology workflows. Recent surveys suggest that these approaches are likely to play an increasingly important role in the development of robust and clinically deployable medical image analysis systems [40,41].

Preliminary comparison with expert manual differential counting demonstrated encouraging agreement between automated segmentation-derived leukocyte quantification and routine microscopy-based assessment. Nevertheless, prospective clinical validation involving larger patient cohorts, direct comparison with flow cytometry, and multi-observer hematological evaluation will remain essential before practical clinical deployment can be considered.

Path Towards Clinical Integration: In a potential deployment setting, TransNet–SAM2 may serve as a computational segmentation component within digital hematology workflows, where whole-slide microscopy images are processed to generate leukocyte masks for subsequent quantitative analysis or expert review. The framework demonstrates computational efficiency (0.08 s per patch), supporting scalable analysis of large microscopy datasets. However, additional prospective validation under routine laboratory conditions will still be necessary before broader clinical integration can be established.

## 5. Conclusions

This study introduced TransNet–SAM2, a hybrid computational framework that combines transformer-based contextual feature learning with foundation-model decoding for automated white blood cell segmentation in microscopic blood smear images. The proposed framework addresses several important challenges in computational hematology image analysis, including dependence on manual prompts, limited cross-dataset generalization, and scarcity of densely annotated training data.

By integrating hierarchical transformer representations with the SAM2 decoder through cross-scale contextual alignment, the framework enables fully automated and prompt-free segmentation while preserving both fine cellular structures and broader spatial context. In addition, the weakly supervised self-training strategy reduces dependence on extensive pixel-level annotations, supporting more practical development of computational pathology systems in clinically constrained settings.

The experimental results demonstrated strong segmentation performance across the Dicle University, Raabin-WBC, and ALL-IDB datasets under varying staining, imaging, and morphological conditions. External validation on leukemic blast-cell images further demonstrated the robustness of the proposed framework under atypical hematological conditions associated with acute lymphoblastic leukemia. Qualitative analysis also showed improved boundary delineation and instance separation compared with the evaluated baseline methods.

Preliminary comparison between automated segmentation-derived leukocyte quantification and expert manual differential counting demonstrated encouraging agreement, providing initial evidence that the proposed framework may support future computer-assisted hematological analysis workflows. Nevertheless, the proposed approach should be considered a supportive computational tool rather than a replacement for expert clinical diagnosis, flow cytometry, or comprehensive hematological interpretation.

Despite the promising performance achieved across the evaluated datasets, several limitations remain. Segmentation accuracy may decrease in regions affected by severe smear artefacts, dense erythrocyte overlap, low cytoplasmic contrast, or highly irregular cellular morphology. Additional challenges arise in cases involving atypical or dysplastic leukemic cells that differ substantially from the training data. Furthermore, the current study does not comprehensively encompass the full spectrum of leukemic subtypes, transitional cellular populations, dysplastic hematological conditions, or bone marrow smear morphology.

These limitations reflect the inherent complexity of automated analysis in real-world hematological microscopy and should be carefully considered during future translational validation studies. Therefore, future work should investigate the proposed framework using larger and more clinically heterogeneous multi-center datasets collected under diverse acquisition conditions. Further studies should focus on improving segmentation robustness in challenging morphological scenarios, incorporating downstream expert-guided classification and diagnostic analysis, and performing broader prospective clinical validation within routine hematological assessment workflows.

In addition, future validation studies should be designed as double-blinded parallel comparative investigations between expert manual microscopic assessment and automated segmentation-based analysis in prospective clinical settings. Such studies should include consecutive peripheral blood smear samples from patients with suspected or confirmed hematological disorders, with both manual differential counts and automated segmentation-derived quantification performed independently and blinded to the results of the alternative assessment method. Important evaluation criteria should include agreement for blast-cell detection, false-negative rates for clinically significant blast populations, time efficiency, reduction in inter-observer variability, and potential impact on diagnostic decision-making. Through such rigorous comparative validation, more comprehensive evidence regarding the relative strengths, limitations, reproducibility, and clinical applicability of computational hematology workflows such as TransNet–SAM2 may be established for routine hematopathology practice.

Overall, the proposed TransNet–SAM2 framework demonstrates the potential of transformer- and foundation-model-based approaches for automated computational analysis of white blood cells within digital hematology and microscopy-assisted analysis workflows.

## Figures and Tables

**Table 1:** Comparative Analysis of Existing Segmentation Frameworks and the Proposed TransNet–SAM2 Architecture.

Method	Global Context	Prompt-Free	Weak Supervision	Foundation Model
U-Net [4]	✗	✓	✗	✗
Trans-UNet [11]	✓	✓	✗	✗
SAM-Adapter [15]	✓	✗	✗	✓
SAM2 [18]	✓	✗	✗	✓
**TransNet-SAM2 (Proposed)**	**✓**	**✓**	**✓**	**✓**

**Note:** ✓ denotes the presence of the corresponding capability within the framework, while ✗ denotes its absence.

**Table 2:** Summary of the datasets used in this study.

Dataset	Image Source	Cell Classes	Patch Size	Annotation Type	Purpose
Dicle University	Peripheral blood smear WSIs	Blast, Neutrophil, Lymphocyte	256 × 256	Pixel-level (QuPath)	Training/Validation/Testing
Raabin-WBC	Public hematology dataset	Multiple WBC types	256 × 256	Pixel-level	Cross-dataset evaluation
ALL-IDB	Public leukemia microscopy dataset	Leukemic blast cells	256 × 256	Pixel-level/Expert annotation	External leukemic blast validation

**Table 3:** Summary of the datasets used in the study.

Dataset	Total Images/Patches	Patch Size	Train	Validation	Test/External Evaluation	Classes
Dicle University	14,720 patches	256 × 256	10,304	2208	2208	Blast, Neutrophil, Lymphocyte
Raabin-WBC	11,200 patches	256 × 256	7840	1680	1680	Lymphocyte, Neutrophil, Monocyte, Eosinophil, Basophil (used as unified foreground)
ALL-IDB	100 images	256 × 256	–	–	100	Leukemic blast cells

**Table 4:** Training hyperparameters used for optimisation of the proposed TransNet–SAM2 framework.

Parameter	Value
Optimizer	Adam
Initial learning rate	1 × 10^−4^
Batch size	8
Number of epochs	60
Patch size	256 × 256
Loss function	BCE + Dice
Learning rate scheduler	Cosine decay

**Table 5:** Segmentation performance comparison on the Dicle University dataset.

Method	Dice	IoU	Precision	Recall
U-Net	0.89 ± 0.01	0.81	0.88	0.90
Mask R-CNN	0.90 ± 0.01	0.83	0.91	0.89
StarDist	0.91 ± 0.01	0.85	0.92	0.90
TransUNet	0.92 ± 0.01	0.86	0.91	0.90
SAM-Adapter	0.93 ± 0.01	0.87	0.94	0.92
SAM2	0.92 ± 0.01	0.86	0.93	0.91
**TransNet–SAM2**	**0.95 ± 0.01**	**0.90**	**0.96**	**0.94**

**Table 6:** Ablation study evaluating the contribution of each architectural component to overall segmentation performance (Dice coefficient).

Configuration	TransNet Backbone	SAM2 Decoder	Cross-Scale Alignment	Self-Training	Dice
Baseline CNN	✗	✗	✗	✗	0.89
Transformer Backbone Only	✓	✗	✗	✗	0.91
TransNet + SAM2	✓	✓	✗	✗	0.93
TransNet + SAM2 + Alignment	✓	✓	✓	✗	0.94
**Full TransNet–SAM2**	✓	✓	✓	✓	✓

**Note:** ✓ indicates that the corresponding component is included in the configuration, whereas ✗ indicates that the component is excluded.

**Table 7:** Effect of loss weighting parameters on segmentation performance.

λ_1_ (BCE)	λ_2_ (Dice)	Dice Score
1.0	0.0	0.91
0.75	0.25	0.93
0.5	0.5	0.95
0.25	0.75	0.94
0.0	1.0	0.92

**Table 8:** Cross-dataset segmentation performance (train: Dicle, test: Raabin-WBC).

Method	Dice	IoU	Precision	Recall
U-Net	0.85	0.76	0.86	0.84
StarDist	0.87	0.79	0.88	0.86
SAM2 baseline	0.88	0.81	0.89	0.87
**TransNet–SAM2 (Proposed)**	**0.91**	**0.84**	**0.92**	**0.90**

**Table 9:** External validation results on leukemic blast cell segmentation using the ALL-IDB dataset.

Method	Dice	IoU	Precision	Recall
U-Net	0.81	0.71	0.83	0.80
StarDist	0.84	0.75	0.85	0.83
SAM2 baseline	0.86	0.78	0.87	0.85
**TransNet–SAM2 (Proposed)**	**0.89**	**0.82**	**0.90**	**0.88**

**Table 10:** Correlation analysis between manual differential counts and TransNet-SAM2 segmentation-derived leukocyte quantification, with breakdown by sample type.

Cell Type	Sample Composition	Pearson r	*p*-Value
Neutrophils	15 peripheral blood smear samples (6 ALL, 4 AML, 5 non-leukemia)	0.93	<0.001
Lymphocytes	15 peripheral blood smear samples (6 ALL, 4 AML, 5 non-leukemia)	0.91	<0.001
Blast Cells	10 leukemia samples (6 ALL, 4 AML)	0.88	<0.01

**Table 11:** Pearson correlation between TransNet–SAM2 automated quantification and flow cytometry reference counts.

Cell Type	Pearson Correlation (r)	*p*-Value
Neutrophils	0.91	<0.01
Lymphocytes	0.89	<0.01
Blast Cells	0.87	<0.05

## Data Availability

The original contributions presented in this study are included in the article. Further inquiries can be directed to the corresponding author.
